# The solution structure of ChaB, a putative membrane ion antiporter regulator from *Escherichia coli*

**DOI:** 10.1186/1472-6807-4-9

**Published:** 2004-08-11

**Authors:** Michael J Osborne, Nadeem Siddiqui, Pietro Iannuzzi, Kalle Gehring

**Affiliations:** 1 Department of Biochemistry, McGill University, 3655 Promenade Sir William Osler, Montreal, Quebec, Canada, H3G 1Y6; 2 Biotechnology Research Institute, National Research Council of Canada, 6100 Royalmount Avenue, Montreal Quebec, Canada, H4P 2R2

**Keywords:** NMR spectroscopy, structure, ChaA, ChaB, antiporter

## Abstract

**Background:**

ChaB is a putative regulator of ChaA, a Na^+^/H^+ ^antiporter that also has Ca^+^/H^+ ^activity in *E. coli*. ChaB contains a conserved 60-residue region of unknown function found in other bacteria, archaeabacteria and a series of baculoviral proteins. As part of a structural genomics project, the structure of ChaB was elucidated by NMR spectroscopy.

**Results:**

The structure of ChaB is composed of 3 α-helices and a small sheet that pack tightly to form a fold that is found in the cyclin-box family of proteins.

**Conclusion:**

ChaB is distinguished from its putative DNA binding sequence homologues by a highly charged flexible loop region that has weak affinity to Mg^2+ ^and Ca^2+ ^divalent metal ions.

## Background

The regulation of cellular ion concentrations is an essential process in all organisms, necessary to sustain a multitude of physiological processes including pH balance and ion homeostasis. This process is accomplished mainly through membrane ion transporters. In *Escherichia coli*, among the membrane proteins that catalyze the exchange of ions across the cell membrane [1] are the Na^+^/H^+ ^antiporters NhaA, NhaB and ChaA, which are involved in sodium ion extrusion. Within *E. coli *and other enteric bacteria, antiporters encompass the primary systems responsible for adaptation to growth in conditions of high Na^+ ^concentrations and varying pH [2-8]. It is common for bacteria to have multiple systems for a similar function. The use of one system is preferred depending on the stress as a means to adapt to varying environmental conditions [9,10]. Of the Na^+^/H^+ ^antiporters, ChaA is unique in that it also shows pH-independent Ca^+^/H^+ ^antiporter activity. ChaA is also regulated by Mg^2+^, which inhibits both its Na^+^/H^+ ^and Ca^+^/H^+ ^antiporter activity [11].

The Cha operon consists of 3 genes, *chaA*, *chaB *and *chaC *found at ~27 minutes on the *E. coli *chromosome [12]. Both ChaB and ChaC are proposed to be regulators of ChaA however, the biological function for either remains to be established. ChaB is a 76-residue protein that contains a conserved 60-residue region found in several other bacteria and baculoviruses. We report here the three-dimensional structure of ChaB determined by NMR spectroscopy and examine key differences between the ChaB families of proteins.

## Results and Discussion

### Assignment of resonances

**Figure 1:**
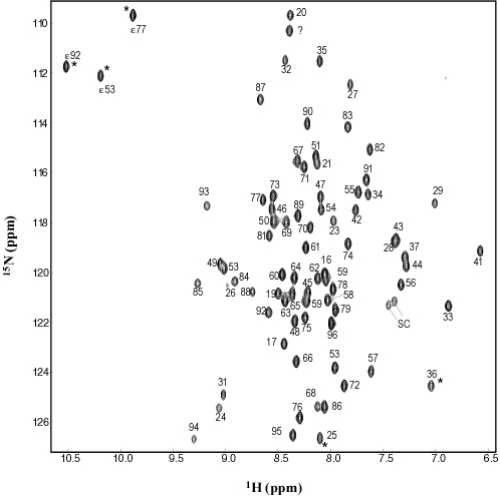
**^1^H-^15^N HSQC spectrum of ChaB at 600 MHz, pH 6.3 in 50 mM CaCl_2_**. Folded resonances are indicated by asterisks. Numbering shown includes the N-terminal His-tag (residues 1–21). The native ChaB sequence starts at P22. The unassigned peak is denoted as a question mark. Folded arginine/lysine side chain resonances are indicated by SC.

**Figure 2:**
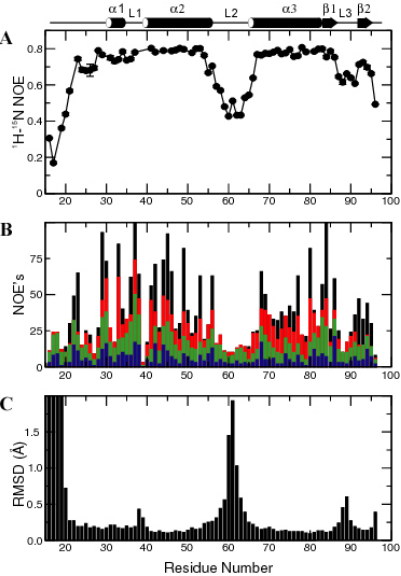
**Plots of ^1^H-^15^N heteronuclear NOE, NOE constraints and RMSD statistics for ChaB. **(A) ^1^H-^15^N heteronuclear NOE acquired at 600 MHz. (B) Summary of all unassigned unambiguous NOE constraints: intra-residue, sequential, medium and long range NOEs are shown as blue, green, red and black bars respectively. (C) Backbone RMSD's calculated for the 17 lowest energy ChaB structures based on superposition of residues P22-S96.

The ^1^H-^15^N HSQC spectrum of ChaB (Fig. 1) is well dispersed suggesting ChaB is a globular, folded protein. Complete ^1^H, ^15^N and ^13^C backbone assignments were made for ChaB, except residues S39 and H40, which yielded no apparent amide cross peaks. Virtually complete assignments (> 98%) were made for the ^1^H, ^13^C, and ^15^N side chain resonances. Resonance assignments have been deposited at BMRB (code 6117). Six signals in the ^1^H-^15^N HSQC spectrum originated from the 21 residue N-terminal His-tag (Fig. 1). The low heteronuclear NOE values (Fig. 2A) and the relatively low number of long range NOE's (Fig. 2B) for these residues indicate the His-tag to be flexible in solution.

### Solution structure of ChaB

**Figure 3:**
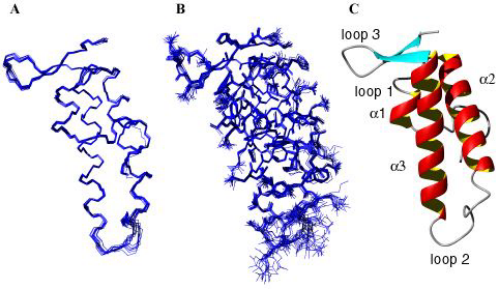
**Solution structure of ChaB**. Ensemble of the 17 lowest energy structures showing (A) backbone and (B) heavy-atom traces. Superposition was made over residues comprising the native ChaB sequence (P22-S96). (C) Ribbon representation of the lowest energy ChaB conformer.

The 3D structure of ChaB (Fig. 3) is well defined by the structural constraints (Table 1) and is dominated by two, relatively long central helices comprising residues H40-Q55 (helix α2) and D65-E83 (helix α3) and a small N-terminal helix (helix α1, E31-K34), which is terminated by a proline (P30). At the C-terminus, a short two-strand β-sheet is observed involving residues Y84-K86 and W92-K94. A tightly packed hydrophobic core stabilizes the overall fold of ChaB. The following hydrophobic residues have < 10% of their surface area exposed to the solvent: Y23, L29, V33, L37, A41, I44, Y45, A48, F49, A52, A72, A76, V80, Y84, A85 and W92. Many of the hydrophobic contacts are between the two long helices (α2 and α3). The C-terminal β-sheet acts as a "cap" for hydrophobic residues from loop 1 (V36, L37), which connects helices α1 and α2, and residues at the N-terminus of helix α2 (A41) and the C-terminus of α3 (V80). Both central helices are largely amphipathic, with residues D43, K46, E47, D54, of helix α2 and E70, K74 and K81 of helix α3 exposed to the bulk solvent and contributing to a highly charged ChaB surface. Most notable, an area of negative charge is observed at the highly mobile loop 2 and the helices immediately surrounding it. In addition, K74, K81, K86, and K95 contribute to a positively charged area while Y56, V75 and A79 present a small hydrophobic patch.

**Table 1:** Constraints and structural statistics for ChaB

Constraints used for structure calculation (all residues)		
Total NOE constraints		2140
Intraresidue NOEs	(n = 0)	515
Sequential NOEs	(n = 1)	432
Medium Range NOEs	(n = 2,3,4)	379
Long Range NOEs	(n > 4)	486
Total Unambigous NOEs		1794
Ambiguous NOE restraints		346
Dihedral angle constraints		49
^15^N-^1^H residual dipolar couplings		58
Average RMSD to mean structure (Å) (residues 22–96)		
Backbone atoms		0.397
All heavy (non-hydrogen atoms)		0.807
Average energy values (kcal mole^-1^) quoted for residues 1–96		
E_total_		-332.42 ± 9.46
E_bond_		11.93 ± 0.89
E_angle_		84.06 ± 1.56
E_improper_		16.35 ± 0.58
E_VdW_		-515.11 ± 10.31
E_NOE_		41.39 ± 3.75
E_dihedral_		0.86 ± 0.21
E_sani_		28.09 ± 3.11
Deviation from idealised covalent geometry		
Bonds (Å)		0.0028 ± 0.0001
Angles (°)		0.4470 ± 0.0042
Improper (°)		0.353 ± 0.006
RMSD from experimental data		
Distance restraints (Å)		0.016 ± 0.0007
Dihedral angle restraints (°)		0.302 ± 0.094
Average Ramachandran statistics for 17 lowest energy structures (residues 22–96)		
Residues in most favored regions		78.2%
Residues in additional allowed regions		8.6%
Residues in generously allowed regions		3.3%
Residues in disallowed regions		0.0%
Analysis of residual dipolar coupling		
RMSD (Hz)		1.495 ± 0.097
Q-factor		0.138 ± 0.0059
Correlation coefficient		0.98

In general, the secondary structure elements of ChaB are well defined exhibiting RMSD's of 0.14 Å and 0.39 Å for backbone and all non-hydrogen atoms, respectively. This is confirmed by the heteronuclear NOE data, which show a 10% trimmed weighted mean of 0.77 ± 0.03 in the structured regions and indicate lack of motions on the nanosecond timescale (Fig. 2A). Regions connecting the secondary structural elements exhibited lower heteronuclear NOE values. In particular, the loops connecting helices α2 and α3 (loop 2, Y56-D64) and the two strands of the β-sheet (loop3, G87-K91) exhibited NOE values below 0.65, indicating large amplitude nanosecond motions in these regions. These motions manifest as regions with large RMSD values in the structural ensemble (Fig. 2C). The small sheet region at the C-terminus was also seen to exhibit some motional freedom particularly for the second strand. However, it is notable that its NOE values are substantially higher than the surrounding loop.

### The ChaB family and structurally similar proteins

**Figure 4:**
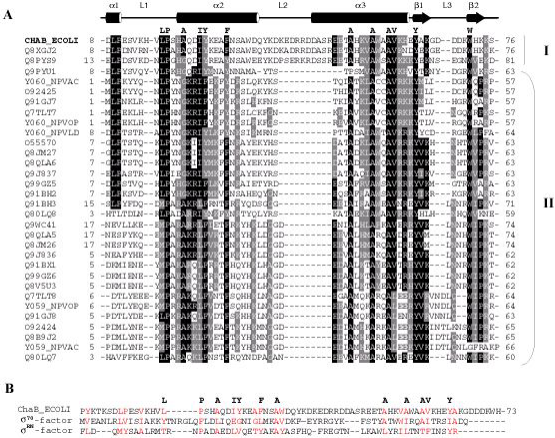
**Sequence alignment of the family of ChaB protein**s. (A) Alignment of ChaB from *E. coli *aligned with a series of related proteins identified by Pfam [34]. In bold above alignments, are residues most conserved among ChaB proteins. Cartoon diagram above represents the secondary structure of ChaB. ChaB from *E. coli *(in bold) *Salmonella typhi *(Q8XGJ2)and *Methanosarcina mazei *(Q8PYS9) are classified as group I ChaB proteins. Group II ChaB proteins are all found in Baculoviridae. The figure was created using BOXSHADE (EMBnet). Identical amino acids are highlighted in black and homologous residues in gray. (B) Sequence alignments of ChaB and related proteins σ^70 ^and σ^RN ^based structural composition (see text for details).

Figure 4 shows the alignment of protein sequences related to *E. coli *ChaB. The most conserved residues make up the hydrophobic core of ChaB, particularly the two long helices and the small sheet. With the exception of P38 and A79 all these residues exhibit < 5% solvent accessibility. These residues are critical for defining the overall fold of ChaB and suggest that all proteins within this family adopt a similar fold.

**Figure 5:**
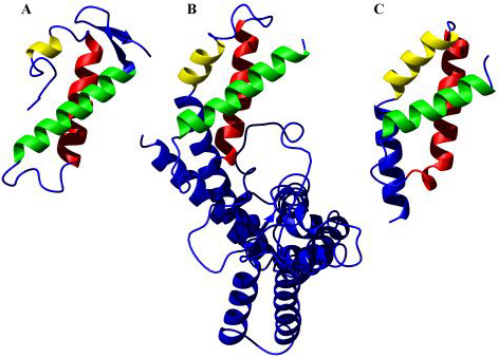
**Comparison of ChaB with structurally similar proteins. **Structural similarities between (A) ChaB, (B) σ^70^(PDB code 1SIG [14]) and (C) σ^RN ^(PDB code 1H3L, [15]) proteins identified from the DALI server. The three helices in ChaB are colour coded, with the equivalent helices in σ^70 ^and σ^RN ^similarly coloured.

Structural homologues of ChaB in the PDB were identified using the DALI server [13] (Fig. 5). This yielded several matches with fragments of other structures, the best match being sigma factor σ^70 ^(PDB code 1SIG, [14]) with a DALI Z-score of 4.7. A DALI-Z score greater than 2.0 is considered structurally similar. Another sigma factor, σ^RN^[15], with little sequence homology to σ^70 ^was identified with a DALI-Z score of 3.5. ChaB, however, exhibits no significant sequence similarity with these proteins. Sigma factors are proteins, which bind to DNA dependent RNA polymerases to form the holeoenzyme [16,17]. Although the observed structural similarities do not define a functional role for ChaB it is worth noting that the σ^RN ^domain is classified in the cyclin-box fold of proteins [15], a class of proteins that bind a diverse set of proteins and nucleic acids [18].

An interesting observation can be made when aligning the sequences of ChaB, σ^70 ^and σ^RN ^based on their structural similarity (Fig. 4B). Residues in the three structures with < 10% of the surface exposed to the solvent are highlighted in red. Clearly, the hydrophobic core, critical for the ChaB fold (marked above the sequence alignments in Fig. 4) is also important for the fold observed in the sigma factors. Key hydrophobic residues appear in similar locations in their "structural space" between the three proteins, forging contacts important for stabilizing the fold. These residues are among the most conserved in the two sigma factor families and within ChaB proteins.

### Loop 2 has weak affinity for divalent ions

**Figure 6:**
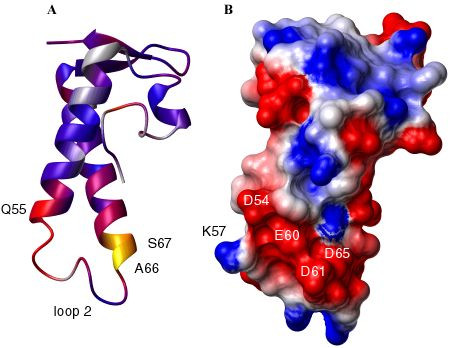
**CaCl_2 _titration and surface potential map of ChaB. **(A) Summary of the perturbations of CaCl_2 _on the backbone ^1^H-^15^N chemical shifts of ChaB. The change in chemical shifts (determined from a weighted vector sum of ^1^H and ^15^N ppm deviations) are mapped onto the structure of ChaB using a colour gradient from blue, to red to yellow, where yellow is the largest perturbation and blue the smallest. Residues that could not be analyzed such as overlapping residues or residues that do not exhibit NH resonances are coloured grey. (B) Potential map of the surface of ChaB calculated using MOLMOL [35] shown in the same orientation as (A). Residues most perturbed by Ca^2+ ^cluster around a highly negatively charged patch on the ChaB surface comprising a flexible loop.

Given the proposed function of ChaB as a regulator and the effect of magnesium as an inhibitor of the Ca^+^/H^+ ^antiporter ChaA, we examined the influence of calcium and magnesium ions on ChaB ^15^N-^1^H chemical shifts. The pattern of perturbed shifts (summarised in Fig. 6A for Ca^2+^) indicates that the highly charged (Fig. 6B) flexible loop 2 and surrounding regions are most important for binding. Chemical shift perturbations of similar magnitude and direction were witnessed upon addition of MgCl_2 _indicating that Mg^2+ ^has a similar binding site and affinity for ChaB as Ca^2+^. The observed association constant for CaCl_2 _is weak (the K_D _was estimated to be > 10 mM by NMR) and not likely to be physiologically significant. Given ChaB's proposed role as a regulatory protein, it is possible that the affinity for Ca^2+ ^or Mg^2+ ^is increased in the presence of ChaA or ChaC.

### A functional role for loop 2?

ChaB proteins are classified into two major groups based on their sequence alignments (Fig. 4). Group I consists of ChaB proteins found in bacteria (*E. coli *and *Salmonella typhi*) and archeabacteria (*Methanosarcina mazei*), while Group II contain ChaB related proteins that are found in Baculoviridae. Thus far, no ChaB domains have been identified in vertebrate and plant species. One major difference between the two classes of ChaB proteins is the presence of the charged loop (loop 2, Fig. 4) that we have shown to bind weakly to Ca^2+^and Mg^2+^ions. The EMBL European bioinformatic database annotates ChaB proteins found within group II (Baculoviridae, Fig. 4) as putative DNA binding proteins. Interestingly, the σ-factor domains that are structurally similar to ChaB are known to bind DNA at the position equivalent to helix α3 in ChaB. However, the composition of the corresponding loop in sigma factors is more hydrophobic and/or shorter than in ChaB. Members of the ChaB sequences belonging to group I (Fig. 4) are annotated as cation transport regulators based on being part of the ChaA operon. The alignment results suggest that the loop 2 region, which is only observed in the group I family of ChaB, is correlated to its function as a cation transport regulator protein. Clearly, further experiments are required to test this hypothesis.

## Conclusion

As part of the *E. coli *structural genomics project, we report the first 3D structure of a member from the ChaB protein family. *E. coli *ChaB is a putative cation transport regulator protein whose structure resembles the cyclin-box fold. ChaB was shown to have weak affinity for calcium and magnesium ions at a highly charged and mobile loop that is only present in ChaB family members associated with a cation transporter. We hypothesise that this loop may play a role in the function of ChaB as a regulator of cation transport.

## Methods

### Cloning, expression and purification of ChaB

The gene encoding full length ChaB (residues 1–76) was amplified from genomic *E. coli *DNA strain O157:H7 using oligos OPI403 AAAAAAGGATCCCCGTATAAAACGAAAAGCGACCTG and OPI499 AAAAAAGAATTCTTACGATTTTTTATGCCATTTATCATCA. Underlined are restriction sites BamHI and EcoRI for both oligos, respectively. The product was cloned into the BamHI/EcoRI site of pFO-1. The plasmid pFO-1 is a tailored pET-15b vector (Novagen Inc., Madison, WI), which contains an extended poly-linker region and an 8× N-terminal histidine tag with a modified thrombin cleavage site.

The ChaB construct (plasmid ID: pPI489) was expressed in *E. coli *BL21-Gold (DE3) cells (Stratagene) as an 8× His-tagged fusion protein. At an OD_600 _of 0.8, the cells were induced with 1 mM IPTG and grown for another 3 hours at 30°C. The protein was purified to homogeneity by absorption onto a Ni^2+ ^charged chelating sepharose column (Amersham Biosciences) under native conditions. The recombinant protein used for NMR studies consists of the ChaB sequence with an extra 21 residues (MGSSHHHHHHHHSSGFNPRGS) at the N-terminus containing the 8× His-tag and a thrombin cleavage site. This tag replaced the first residue, a methionine, of the genomic sequence of ChaB. In the analysis *vide ante *ChaB refers to residues P22-S96 of our construct (i.e. the wild type ChaB sequence excluding the first Met residue). Thus, ChaB begins at P22 in our numbering scheme, corresponding to P2 in the native ChaB sequence. The mass of ChaB was confirmed by SDS-PAGE and electrospray mass spectroscopy.

### NMR Spectroscopy

Uniform enrichment of ChaB with ^15^N and/or ^13^C was achieved by growing the bacteria in M9 minimal medium supplemented with BME vitamins (SIGMA) and (^15^NH_4_)_2_SO_4 _and/or ^13^C_6_-glucose as the sole nitrogen and carbon sources at 37°C. ChaB was purified as described above. NMR samples were obtained by exchanging ChaB into an NMR buffer comprising 50 mM CaCl_2 _at pH 6.3 using a PD-10 column and subsequent concentration to ~200–300 μL using an Amicon Ultra-4 (5 KDa cutoff, Millipore). Typical protein concentrations ranged from 1.5–2.0 mM.

NMR spectra for resonance assignments were recorded at 303 K on a Bruker Avance DRX 600 MHz spectrometer equipped with a triple-resonance CryoProbe and processed with NMRPipe [21]. Backbone ^1^H, ^13^C and ^15^N assignments were completed from CBCA(CO)NH, CBCANH and HBHA(CBCACO)NH spectra using a combination of NMRView [22] and SMARTNOTEBOOK (a module designed for semi-automated assignment in NMRView) [23] packages. ^1^H, ^13^C and ^15^N sidechain assignments were obtained by manual analysis of the H(CC)(CO)NH, C(C)(CO)NH and HCCH-TOCSY experiments using NMRView and in-house written scripts. ^1^H, ^13^C and ^15^N chemical shifts were referenced to DSS according to the IUPAC recommendation [24].

Distance constraints were obtained from a simultaneous 3D ^13^C/^15^N-edited NOESY experiment (τ_m_= 120 ms) in 90% H_2_O/10% D_2_O, and ^13^C-edited NOESY (τ_m _= 100 ms) and ^13^C-edited NOESY (aromatic region) (τ_m _= 100 ms) experiments acquired in 99.9% D_2_O. The experiments in D_2_O were acquired at 800 MHz on a Varian INOVA spectrometer at NANUC). A 4D ^13^C-^13^C edited NOESY experiment (τ_m _= 100 ms) was acquired at 600 MHz to resolve ambiguities involving methyl groups. For all experiments at 600 MHz, the minimal number of scans dictated by the phase cycle was used in combination extensive folding in ^15^N and ^13^C to reduce experimentation time. Additional restraints used in structure calculations were: dihedral restraints, derived from ^3J^HN-Cα coupling constants obtained from the HNHA experiment [25] and ^1^H-^15^N residual dipolar couplings extracted from comparison of IPAP-HSQC experiments recorded on ChaB with and without 11 mg/mL Pf1 phage [26]. For the measurement of dipolar couplings, the NMR buffer was altered to 50 mM phosphate and 100 mM NaCl, pH 6.3 since the presence of CaCl_2 _precipitated the Pf1 phage.

Steady state {^1^H}-^15^N NOE spectra were acquired in an interleaved manner in which each individual FID was collected with and without presaturation and a recycle delay of 4 s [27]. Saturation was achieved using a train of 120° pulses separated by 5 ms for a total irradiation time of 3 s.

### Structure calculations

A set of unambiguous NOE constraints were extracted from the 3D-NOESY spectra and used in conjunction with dihedral angle restraints to generate a preliminary fold of ChaB using CNS1.1 [28]. The resulting structures were used as model templates for automated assignment of NOE peaks using the ARIA 1.1 package [29]. In many cases, the 4D ^13^C-^13^C NOESY experiment was important for manually assigning a number of ambiguous assignments. A total of 1794 unambiguous and 346 ambiguous NOE restraints were obtained from this method and used in combination with dihedral restraints to calculate an ensemble of ChaB structures using CNS [28]. These structures were further refined using residual dipolar coupling restraints. The axial and rhombic components of the alignment tensor were obtained from the histogram method [30] and optimized by a grid search [31] and determined to be D_a _= 13.7 and R = 0.325. Only residues exhibiting a heteronuclear NOE > 0.65 were included as residual dipolar couplings. Seventeen lowest energy structures with the fewest violations were selected to represent the ChaB structure. No NOE violations over 0.2Å were observed. Structural statistics for this ensemble as calculated by CNS [28], PROCHECK [32] and SSIA [33] are summarised in Table 1. The coordinates have been deposited in the RCSB under PDB code 1SG7.

### Titration with calcium and magnesium

The effect of calcium on ChaB was determined from addition of aliquots of 5 M CaCl_2 _or 2 M MgCl_2 _to ^15^N labeled ChaB. Prior to titration, metal impurities were removed by addition of EDTA to the ChaB sample followed by exchange into 20 mM Bis-Tris buffer, pH 6.3 using a PD-10 column. Aliquots of CaCl_2 _or MgCl_2 _were added up to a final concentration of 50 mM. Minimal changes in pH and volume were ensured throughout. Chemical shift perturbations were measured as a weighted vector sum of the ^1^H and ^15^N chemical deviations: {[(Δ^1^H ppm)^2 ^+ (Δ^15^N ppm × 0.2)^2^]^0.5^}.

## List of abbreviations

NMR: nuclear magnetic resonance NOE: nuclear Overhauser enhancement HSQC: heteronuclear single quantum coherence PPM: parts per million RMSD: root mean squared deviation PDB: Protein Data Bank

## Authors' contributions

MJO expressed and purified isoptically enriched ChaB, collected all NMR spectra at 600 MHz, processed and analyzed NMR data, performed structural calculations and structural refinement. NS identified ChaB among a series of *E. coli *proteins cloned as part of the structural genomics initiative. PI completed the initial cloning of chaB. NS expressed, purified and characterized ChaB by mass spectrometry. MJO drafted and NS contributed to the written manuscript. KG coordinated and provided financial support for this study.

## References

[B1] Ambudkar SV, Rosen BP (1990). Bacterial Energetics.

[B2] Padan E, Gerchman Y, Rimon A, Rothman A, Dover N, Carmel-Harel O (1999). The molecular mechanism of regulation of the NhaA Na+/H+ antiporter of Escherichia coli, a key transporter in the adaptation to Na+ and H+. Novartis Found Symp.

[B3] Padan E, Schuldiner S (1993). Na+/H+ antiporters, molecular devices that couple the Na+ and H+ circulation in cells. J Bioenerg Biomembr.

[B4] Padan E, Schuldiner S (1994). Molecular physiology of Na+/H+ antiporters, key transporters in circulation of Na+ and H+ in cells. Biochim Biophys Acta.

[B5] Padan E, Venturi M, Gerchman Y, Dover N (2001). Na(+)/H(+) antiporters. Biochim Biophys Acta.

[B6] Sakuma T, Yamada N, Saito H, Kakegawa T, Kobayashi H (1998). pH dependence of the function of sodium ion extrusion systems in Escherichia coli. Biochim Biophys Acta.

[B7] Boot IR, Cash P, O'Byrne C (2002). Sensing and adapting to acid stress. Antonie Van Leeuwenhoek.

[B8] Konings WN, Albers SV, Koning S, Driessen AJ (2002). The cell membrane plays a crucial role in survival of bacteria and archaea in extreme environments. Antonie Van Leeuwenhoek.

[B9] Kobayashi H, Saito H, Kakegawa T (2000). Bacterial strategies to inhabit acidic environments. J Gen Appl Microbiol.

[B10] Shijuku T, Yamashino T, Ohashi H, Saito H, Kakegawa T, Ohta M, Kobayashi H (2002). Expression of chaA, a sodium ion extrusion system of Escherichia coli, is regulated by osmolarity and pH. Biochim Biophys Acta.

[B11] Ivey DM, Guffanti AA, Zemsky J, Pinner E, Karpel R, Padan E, Schuldiner S, Krulwich TA (1993). Cloning and characterization of a putative Ca2+/H+ antiporter gene from Escherichia coli upon functional complementation of Na+/H+ antiporter-deficient strains by the overexpressed gene. J Biol Chem.

[B12] Oshima T, Aiba H, Baba T, Fujita K, Hayashi K, Honjo A, Ikemoto K, Inada T, Itoh T, Kajihara M (1996). A 718-kb DNA sequence of the Escherichia coli K-12 genome corresponding to the 12.7–28.0 min region on the linkage map (supplement). DNA Res.

[B13] Holm L, Sander C (1995). Dali: a network tool for protein structure comparison. Trends Biochem Sci.

[B14] Malhotra A, Severinova E, Darst SA (1996). Crystal structure of a sigma 70 subunit fragment from E. coli RNA polymerase. Cell.

[B15] Li W, Stevenson CE, Burton N, Jakimowicz P, Paget MS, Buttner MJ, Lawson DM, Kleanthous C (2002). Identification and structure of the anti-sigma factor-binding domain of the disulphide-stress regulated sigma factor sigma(R) from Streptomyces coelicolor. J Mol Biol.

[B16] Burgess RR, Travers AA, Dunn JJ, Bautz EK (1969). Factor stimulating transcription by RNA polymerase. Nature.

[B17] Travers AA, Burgessrr (1969). Cyclic re-use of the RNA polymerase sigma factor. Nature.

[B18] Noble ME, Endicott JA, Brown NR, Johnson LN (1997). The cyclin box fold: protein recognition in cell-cycle and transcription control. Trends Biochem Sci.

[B19] Skelton NJ, Kordel J, Forsen S, Chazin WJ (1990). Comparative structural analysis of the calcium free and bound states of the calcium regulatory protein calbindin D9K. J Mol Biol.

[B20] Skelton NJ, Kordel J, Akke M, Chazin WJ (1992). Nuclear magnetic resonance studies of the internal dynamics in Apo, (Cd2+)1 and (Ca2+)2 calbindin D9k. The rates of amide proton exchange with solvent. J Mol Biol.

[B21] Delaglio F, Grzesiek S, Vuister GW, Zhu G, Pfeifer J, Bax A (1995). NMRPipe: a multidimensional spectral processing system based on UNIX pipes. J Biomol NMR.

[B22] Johnson BA, Blevins RA (1994). NMRView: A computer program for the visualization and analysis of NMR data. Journal of Biomolecular NMR.

[B23] Slupsky CM, Boyko RF, Booth VK, Sykes BD (2003). Smartnotebook: a semiautomated approach to protein sequential NMR resonance assignments. J Biomol NMR.

[B24] Markely JL, Bax A, Arata Y, Hilbers CW, Kaptein R, Sykes BD, Wright PE, Wutrich K (1998). Recommendations for the presentaion of NMR structures of proteins and nucleic acids. Pure & Applied Chemistry.

[B25] Kuboniwa H, Grzesiek S, Delaglio F, Bax A (1994). Measurement of HN-H alpha J couplings in calcium-free calmodulin using new 2D and 3D water-flip-back methods. J Biomol NMR.

[B26] Ottiger M, Bax A (1998). Characterization of magnetically oriented phospholipid micelles for measurement of dipolar couplings in macromolecules. J Biomol NMR.

[B27] Farrow NA, Muhandiram R, Singer AU, Pascal SM, Kay CM, Gish G, Shoelson SE, Pawson T, Forman-Kay JD, Kay LE (1994). Backbone dynamics of a free and phosphopeptide-complexed Src homology 2 domain studied by 15 N NMR relaxation. Biochemistry.

[B28] Brunger AT, Adams PD, Clore GM, DeLano WL, Gros P, Grosse-Kunstleve RW, Jiang JS, Kuszewski J, Nilges M, Pannu NS (1998). Crystallography & NMR system: A new software suite for macromolecular structure determination. Acta Crystallogr D Biol Crystallogr.

[B29] Nilges M, Macias MJ, O'Donoghue SI, Oschkinat H (1997). Automated NOESY interpretation with ambiguous distance restraints: the refined NMR solution structure of the pleckstrin homology domain from beta-spectrin. J Mol Biol.

[B30] Clore GM, Gronenborn AM, Bax A (1998). A robust method for determining the magnitude of the fully asymmetric alignment tensor of oriented macromolecules in the absence of structural information. J Magn Reson.

[B31] Clore GM, Gronenborn AM, Tjandra N (1998). Direct structure refinement against residual dipolar couplings in the presence of rhombicity of unknown magnitude. J Magn Reson.

[B32] Laskowski RA, Rullmannn JA, MacArthur MW, Kaptein R, Thornton JM (1996). AQUA and PROCHECK-NMR: programs for checking the quality of protein structures solved by NMR. J Biomol NMR.

[B33] Zweckstetter M, Bax A (2000). Prediction of sterically induced alignment in a dilute liquid crystalline phase: aid to protein strucutre determination by NMR. Journal of the American Chemical Society.

[B34] Bateman A, Birney E, Cerruti L, Durbin R, Etwiller L, Eddy SR, Griffiths-Jones S, Howe KL, Marshall M, Sonnhammer EL (2002). The Pfam protein families database. Nucleic Acids Res.

[B35] Koradi R, Billeter M, Wuthrich K (1996). MOLMOL: a program for display and analysis of macromolecular structures. J Mol Graph.

